# Validation of Sentinel-3A/3B Satellite Altimetry Wave Heights with Buoy and Jason-3 Data

**DOI:** 10.3390/s19132914

**Published:** 2019-07-01

**Authors:** Jungang Yang, Jie Zhang

**Affiliations:** The First Institute of Oceanography, Ministry of Natural Resources of China, Qingdao 266061, China

**Keywords:** SentinEl-3A, Sentinel-3B, SRAL, significant wave height, data validation

## Abstract

The validation of significant wave height (SWH) data measured by the Sentinel-3A/3B SAR Altimeter (SRAL) is essential for the application of the data in ocean wave monitoring, forecasting and wave climate studies. Sentinel-3A/3B SWH data are validated by comparisons with U. S. National Data Buoy Center (NDBC) buoys, using a spatial scale of 25 km and a temporal scale of 30 min, and with Jason-3 data at their crossovers, using a time difference of less than 30 min. The comparisons with NDBC buoy data show that the root-mean-square error (RMSE) of Sentinel-3A SWH is 0.30 m, and that of Sentinel-3B is no more than 0.31 m. The pseudo-Low-Resolution Mode (PLRM) SWH is slightly better than that of the Synthetic Aperture Radar (SAR) mode. The statistical analysis of Sentinel-3A/3B SWH in the bin of 0.5 m wave height shows that the accuracy of Sentinel-3A/3B SWH data decreases with increasing wave height. The analysis of the monthly biases and RMSEs of Sentinel-3A SWH shows that Sentinel-3A SWH are stable and have a slight upward trend with time. The comparisons with Jason-3 data show that SWH of Sentinel-3A and Jason-3 are consistent in the global ocean. Finally, the piecewise calibration functions are given for the calibration of Sentinel-3A/3B SWH. The results of the study show that Sentinel-3A/3B SWH data have high accuracy and remain stable.

## 1. Introduction

Ocean waves are disturbances in the ocean that transmit energy from one place to another. Ocean waves are initiated where wind and water interact, and then travel across the sea until they collapse on the shore. Ocean waves can travel thousands of miles before reaching land. Long-term variations in ocean waves are related to global climate change. Ocean wave data over long-term periods are necessary for estimating the wave climate and sea state [1,2,3]. Because in situ observations of ocean waves are sparse in space and time and expensive, global ocean wave data are generally derived from satellite observations or ocean wave modelling. Satellite altimetry has enabled a global observation of ocean wave. After the emergence of the satellite altimeter, the global monitoring of ocean wave became a reality. The first global atlas of SWH data derived from GEO-3 altimeter data was published [4]. Since then, SWH data from many different satellites have become available, including SEASAT (1978), GEOSAT (1985–1990), TOPEX/POSEIDON (1992–2006), ERS-1 (1991–2000), ERS-2 (1995–2011), ENVISAT (2002–2012), Cryosat-2 (2010–present), SARAL/AltiKa (2013–present), Jason-1 (2002–2013), Jason-2 (2008–present), Jason-3 (2016–present), HY-2A (2011–present), Sentinel-3A (2016–present) and Sentinel-3B (2018–present).

The Sentinel-3 mission is jointly operated by European Space Agency (ESA) and the European Organization for the Exploitation of Meteorological Satellites (EUMETSAT) to deliver operational ocean and land observation services. The Sentinel-3 mission as part of the Copernicus Programme Copernicus is a constellation of the European Earth Observation satellite mission. There are currently two satellites—Sentinel-3A and 3B in orbit. The main objective of the Sentinel-3 mission is to measure sea surface topography, global surface temperature, and global surface color with high accuracy and reliability to support ocean forecasting systems, environmental monitoring and climate monitoring. The Sentinel-3A and -3B satellites were launched on 16 February 2016 and 25 April 2018, respectively [5].

A SAR Altimeter (SRAL) instrument is equipped on Sentinel-3A/3B to derive sea surface height (SSH), SWH and surface wind speed over the global ocean to an accuracy and precision equivalent to that formerly achieved by ENVISAT Radar Altimeter-2 (RA-2), but with enhanced sea surface height measurements in the coastal zone, sea ice regions and over inland rivers and lakes. Two modes of SWH measurement are supplied by SRAL: Low-Resolution Mode (LRM) and Synthetic Aperture Radar (SAR) mode.

Long-term satellite altimeter SWH data with high accuracy are very important to global ocean wave and climate change studies. Data validation is a necessary step in developing a quality-controlled and fully calibrated and validated satellite dataset. The validation and calibration of satellite altimetry wave heights have been carried out in many studies. Buoy data are generally assumed to be of high quality and have been used in numerous studies for the validation of altimeter wave height data. Wave height data from satellite altimeters have been validated against buoy observations [6,7,8,9,10] and operational wave models [11,12,13]. In addition, SWH data have been cross-validated by different satellite altimeters [14,15,16,17].

The main objectives of this study are to examine the performance of Sentinel-3A/3B SRAL in ocean wave observation and to validate Sentinel-3A/3B SRAL SWH data via comparison with U. S. National Data Buoy Center (NDBC) buoy data and with Jason-3. In addition, SWH observations of Sentinel-3A/3B are calibrated. Section 2 presents data and methods. Section 3 reveals the results and discusses the comparisons of Sentinel-3A/3B SWH with NDBC buoy data and Jason-3. Section 4 presents the conclusions.

## 2. Data and Methods

### 2.1. Sentinel-3A/3B Data

Sentinel-3A and Sentinel-3B are sun-synchronous polar-orbiting satellites operating at a mean altitude of 815 km and inclination of 98.6°. Sentinel-3B’s orbit is identical to that of Sentinel-3A but it flies 140° out of phase with Sentinel-3A. Sentinel-3A/3B has a 27-day repeat cycle to provide global sea surface height, significant wave height and wind speed measurement data with a maximum inter-track distance of 104 km at the equator. The SRAL instrument is a dual-frequency (Ku and C-band) nadir-looking radar altimeter that employs SAR altimetry technologies inherited from the CryoSat-2. The technical characteristics of the Sentinel-3 SRAL instrument are shown in Table 1. The Sentinel-3 mission is also equipped with a microwave radiometer for wet troposphere correction and an on-board precise orbit determination (POD) system for accurate orbit determination. The measurement modes of SRAL instrument are composed of two radar modes which are Low-Resolution Mode (LRM) and SAR mode. LRM is a conventional altimeter pulse-limited mode and SAR mode is a high along-track resolution mode [18]. However, as only one operational mode can be used at a given time, and as the ability to emulate LRM from SAR mode is developed by the reduced SAR mode technique, which results in pseudo-LRM (PLRM), Sentinel-3A/3B operates all over the globe in SAR mode only. Sentinel-3A/3B SAR Radar Altimeter Level 2 Non-Time-Critical (NTC) data, which are distributed by the European Organization for the Exploitation of Meteorological Satellites (EUMETSAT) through Copernicus Online Data Access (https://eoportal.eumetsat.int/userMgmt/login.faces (after 20 January 2018), https://codarep.eumetsat.int/#/home (before 20 January 2018)), are used in this study. The complete product contains three NetCDF files which are reduced measurement, standard measurement and enhanced measurement data [19]. The standard measurement data of SRAL products are used in this study. Considering the data integrity of the whole cycle, the data period of Sentinel-3A SRAL is from 13 March 2016 to 25 February 2019, which covers Cycles 2–41 of SRAL. The data period of Sentinel-3B SRAL is from 10 November 2018 to 7 March 2019 which covers Cycles 17–22 of SRAL. Only Ku-band SAR and PLRM mode SWH data values of no more than 8 m with surface_type_01 flag value 0 (0 for open oceans or semi-enclosed seas) and swh_ocean_qual_01_ku flag value 0 (0 for good), simultaneously, are selected in the study.

**Figure 1:**
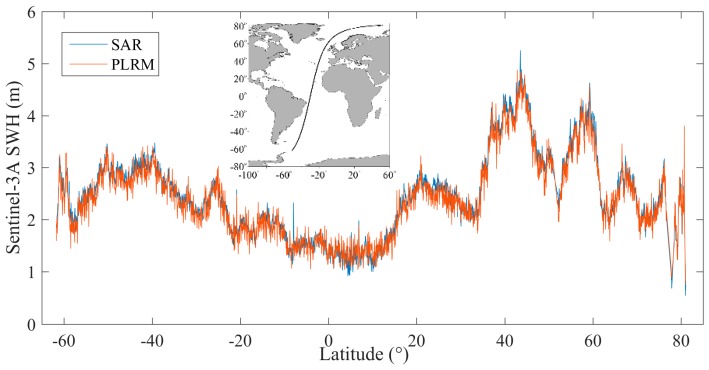
Ku-band SAR and pseudo-low-resolution mode (PLRM) SWH along Sentinel-3A SRAL Cycle 23 pass 237.

**Figure 2:**
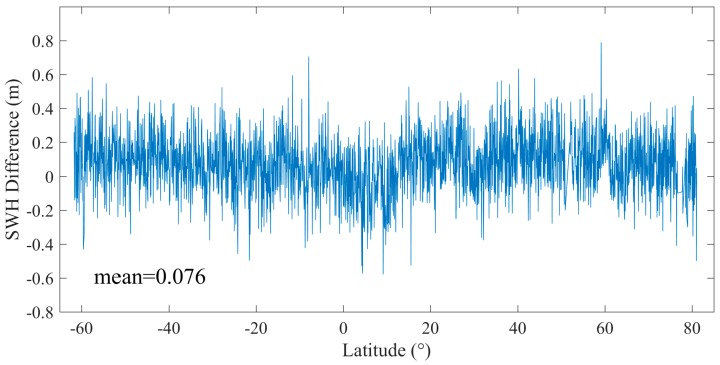
SWH difference between SAR and PLRM modes along Sentinel-3A SRAL Cycle 23 pass 237.

**Figure 3:**
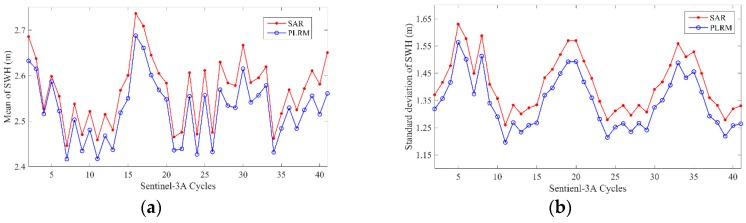
Cycle mean (**a**) and standard deviation (**b**) of Sentinel-3A SAR and PLRM mode SWH.

Figure 1 is an example of Sentinel-3A SWH distribution along Pass 237 of Cycle 23, whose location is shown in Figure 1. It is shown that there is a small SWH difference between SAR and PLRM modes, and SAR mode SWH is slightly larger than that of PLRM mode. The SWH difference between the two modes is shown in Figure 2 and the mean SWH difference is about 0.08 m. The cycle mean and the standard deviation of SWH for the two modes of Sentinel-3A are given in Figure 3. It can be seen that the cycle mean and standard deviation of SAR mode are higher than those of PLRM mode. It is shown that the consistency and difference in Ku-band SWH exist between SAR and PLRM modes.

### 2.2. Jason-3 Data

Jason-3 Geophysical Data Records (GDRs) data distributed by Centre national d’études spatiales (CNES) Archiving, Validation and Interpretation of Satellite Oceanographic Data (AVISO; ftp://avisoftp.cnes.fr) during the same period as the Sentinel-3A data, from 20 December 2016 to 10 February 2019 (cycles 32–74), are used in this study. Jason-3 is an international cooperative satellite altimeter mission between the National Aeronautics and Space Administration (NASA), National Oceanic and Atmospheric Administration (NOAA), EUMETSAT and CNES. Jason-3, which was launched on 17 January 2016, is the successor to the Jason-2, Jason-1 and T/P missions. These altimeters measured ocean surface topography, SWH and wind speed from 1992 to the present. These satellites’ mission is to provide a unique global view of the oceans and to supply data for scientific and practical applications related to sea level rise, ocean circulation and climate change studies. The Jason-3 satellite altimeter operates at Ku and C bands. SWH data of the Ku-band are used in the validation of Sentinel-3A SWH data, by comparisons between Sentinel-3A and Jason-3 at their dual-crossovers.

### 2.3. Buoy Data

**Figure 4:**
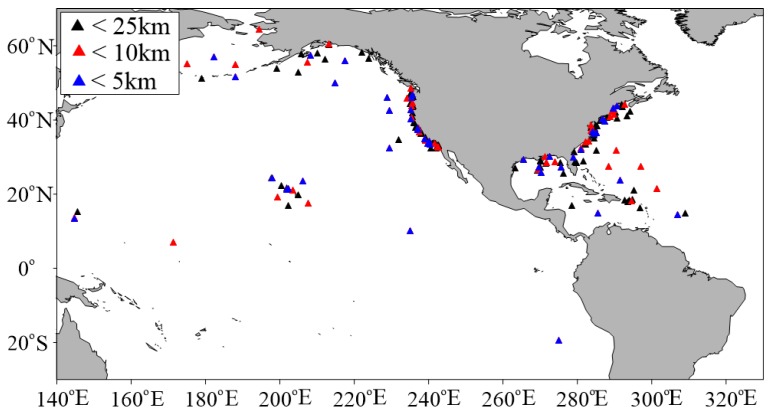
Location of the collocated U. S. National Data Buoy Center (NDBC) buoys with Sentinel-3A.

**Figure 5:**
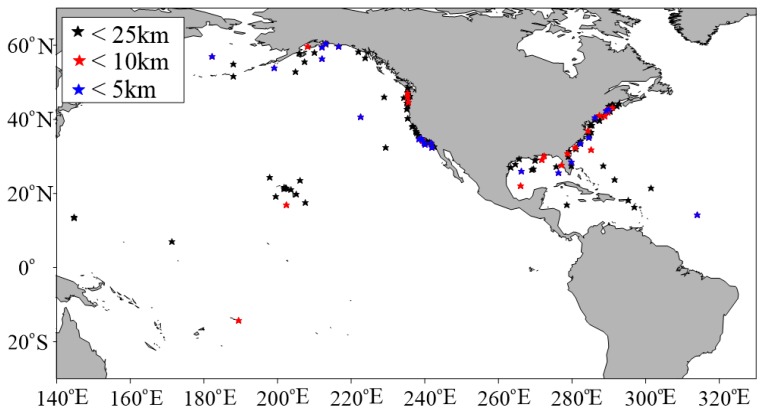
Location of the collocated NDBC buoys with Sentinel-3B.

Wave height data of buoys from the U. S. National Data Buoy Center (NDBC) are used in the validation of Sentinel-3A/3B SWH data. NDBC is a part of the National Oceanic and Atmospheric Administration’s (NOAA) National Weather Service (NWS), and maintains a network of data collecting buoys and coastal stations. Parts of NDBC buoys can measure wave height. NDBC buoy data can be accessed freely (https://www.ndbc.noaa.gov/). All the available NDBC buoy data are used, without taking into account the water depth. To eliminate interference from land, buoys were required to be more than 50 km offshore. NDBC buoy wave height measurements are available hourly from 20-min-long records, or every 10 or 30 min. The collocated NDBC buoys with Sentinel-3A and -3B are shown in Figure 4 and Figure 5. The color means the distance of buoys to the nearest Sentinel-3A or Sentinel-3B measurement point. The blue is less than 5 km, the red is larger than 5 km and less than 10 km, the black is larger than 10 km and less than 25 km.

### 2.4. Validation Method

Sentinel-3A/3B SWH data are validated by comparison with NDBC buoy data and comparison at the dual-crossovers between Sentinel-3A and Jason-3. The ideal comparison should be made using only those sets that are coincident in time and space. However, buoys and altimeters are sampling different aspects of the temporally and spatially varying wave field [22], so, a SWH comparison between Sentinel-3A/3B and NDBC buoys would exhibit differences. Although the criteria of 50 km and 30 min are widely applied [23,24,25], for a better comparison between Sentinel-3A/3B and NDBC buoy data, we use a particular criterion of 25 km and 30 min for temporal and spatial separation by considering the footprint size of Sentinel-3A/3B. Under this criterion, the Sentinel-3A/3B measurement data of the closest distance (less than 25 km) and the minimum observation time difference (less than 30 min) to buoys are collocated. For the comparison at the dual-crossovers between Sentinel-3A and Jason-3, ground-track crossover points are selected when the time difference between the two altimeter pass measurements is less than one given length of time. The crossover point and its SWH data are obtained by the bilinear interpolation of four points at the two altimeter passes which are closest to the crossover point. Then, Sentinel-3A/3B SWH data are evaluated by the statistical analysis of SWH differences.

The statistical parameters used for the validation of Sentinel-3A/3B SWH data are the bias, root-mean-square error (RMSE), scattering index (SI) and correlation coefficient (R), defined as follows:

(1) $$\mathrm{Bias}=\frac{1}{N}\overset{N}{\underset{i\;=\;1}{\sum }}\left(A_{i}-B_{i}\right)$$

(2) $$\mathrm{RMSE}=\sqrt{\frac{1}{N}\overset{N}{\underset{i\;=\;1}{\sum }}{\left(A_{i}-B_{i}\right)}^{2}\;}$$

(3) $$\mathrm{SI}=\frac{\sqrt{\frac{1}{N}\sum_{i\;=\;1}^{N}{\left[\left(A_{i}-\bar{A}\right)-\left(B_{i}-\bar{B}\right)\right]}^{2}}}{\bar{B}}$$

(4) $$R=\frac{\sum_{i\;=\;1}^{N}\left(A_{i}-\bar{A}\right)\left(B_{i}-\bar{B}\right)}{\sqrt{\sum_{i\;=\;1}^{N}{\left(A_{i}-\bar{A}\right)}^{2}{\left(B_{i}-\bar{B}\right)}^{2}}}$$

where *A_i_* represents the SWH from the Sentinel-3A/3B, *B_i_* represents the SWH obtained from the NDBC buoy measurements or Jason-3, *N* is the number of collocated data pairs and the overbar represents the mean value.

## 3. Results and Discussion

### 3.1. Comparison with Buoy Data

**Figure 6:**
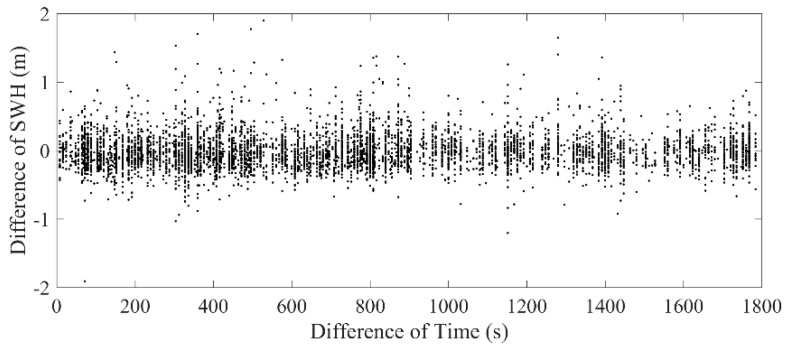
SWH difference of the collocated Sentinel-3A and buoy data at different observation time differences.

According to the validation method introduced in Section 2.4, Sentinel-3A/3B SRAL SWH data are collocated with NDBC buoy data using the spatial and temporal scale of 25 km and 30 min. In order to obtain more collocated data pairs for the Sentinel-3A/3B SWH validation, the SWH differences of the collocated Sentinel-3A/3B and NDBC buoy data and their biases and RMSEs are analyzed with different spatial and temporal scales. The SWH differences of the collocated data with different observation time differences are shown in Figure 6. It is shown that the SWH differences of the collocated data are similar at the different time differences, so a temporal scale of 30 min is selected to obtain more collocated data for the validation of Sentinel-3A/3B SWH data.

**Figure 7:**
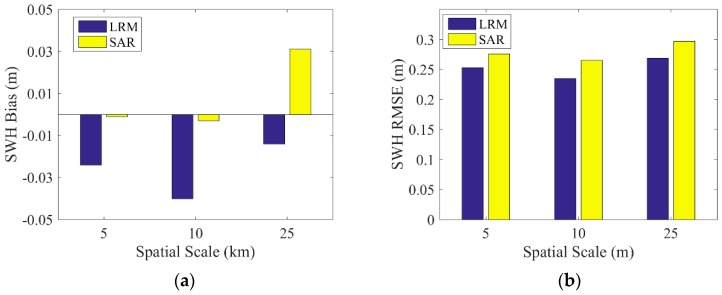
(**a**) Bias and (**b**) root-mean-square error (RMSE) of SWH difference between Sentinel-3A and buoy data at different spatial scales.

**Figure 8:**
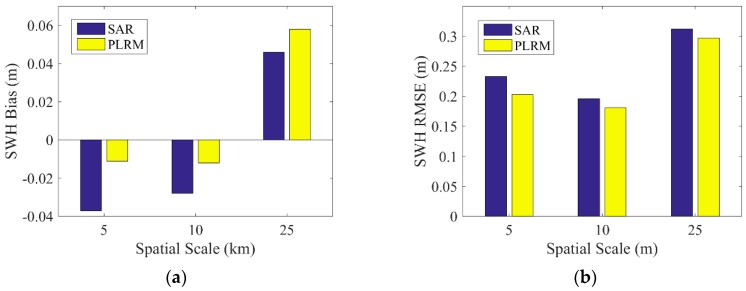
(**a**) Bias and (**b**) RMSE of SWH difference between Sentinel-3B and buoy data at different spatial scales.

For the spatial scale of the data collocation, the spatial scales of 25, 10 and 5 km are selected to obtain the collocated data of Sentinel-3A/3B and NDBC buoys. The biases and RMSEs of two modes of SWH data from Sentinel-3A/3B and NDBC buoy data in three cases of different spatial scales are shown in Figure 7 and Figure 8. For the comparison of Sentinel-3A SWH with buoy data, the bias of Sentinel-3A SAR mode SWH is slightly larger than that of PLRM mode, and the bias of SAR mode shows no trend with increasing spatial scale, while the bias of PLRM mode increases with increasing spatial scale. The RMSEs of Sentinel-3A SAR and PLRM modes increase with increasing spatial scale, generally. The value of RMSE is about 0.2–0.3 m. for the comparison of Sentinel-3B SWH with buoy data, the biases are −0.04–0.06 m and there is no trend with increasing spatial scale. The RMSEs for Sentinel-3B are about 0.18–0.3 m and show a small increase with increasing spatial scale. In general, there is no obvious difference in statistical parameters across different spatial scales of data collocation. So, the collocated data of the spatial scale of 25 km are selected to obtain more collocated data for the validation of Sentinel-3A/3B SWH data.

**Figure 9:**
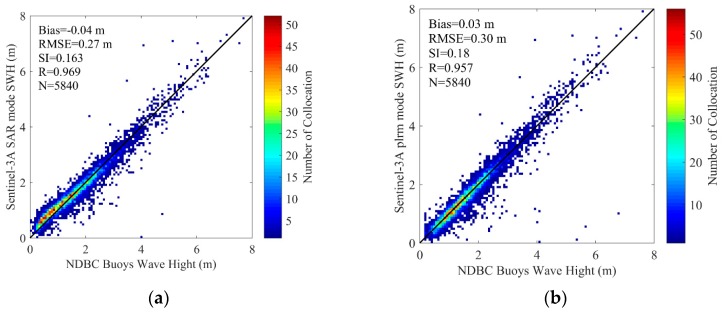
Scatterplots for the SWH comparisons between Sentinel-3A (**a**) SAR and (**b**) PLRM modes and NDBC buoys.

**Figure 10:**
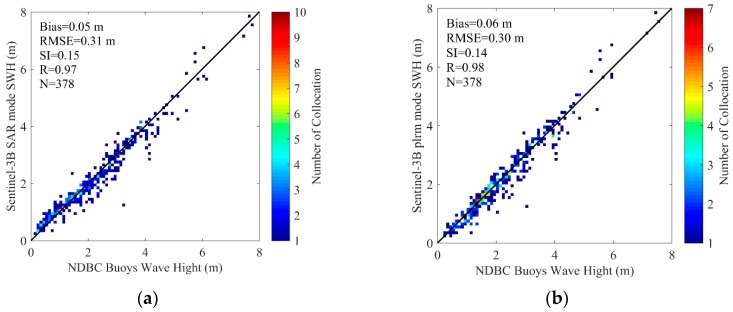
Scatterplots for the SWH comparisons between Sentinel-3B (**a**) SAR and (**b**) PLRM modes and NDBC buoys.

With the spatial scale of 25 km and temporal scale of 30 min, 5840 collocated data pairs of 172 NDBC buoys (as shown in Figure 4) for Sentinel-3A SWH data comparison, and 378 collocated data pairs of 123 NDBC buoys (as shown in Figure 5) for Sentinel-3B SWH data comparison, are obtained. The scatter diagrams of the comparison between Sentinel-3A/3B SAR and PLRM mode SWH are shown in Figure 9 and Figure 10. The statistical parameters are calculated and given in these figures. The bias, RMSE, scattering index (SI) and correlation coefficient (R) of Sentinel-3A SAR mode SWH are 0.04 m, 0.27 m, 0.16 and 0.97, and those of Sentinel-3A PLRM mode SWH are 0.03 m, 0.30 m, 0.18 and 0.96. The similar SI and R values for Sentinel-3A SAR and PLRM mode SWH show the consistency of Sentinel-3A SWH and buoy data. The statistical parameters of Sentinel-3B SWH also present the same result. For Sentinel-3A, the bias of SAR mode is slightly larger than that of PLRM mode, and the RMSE of SAR mode is lower than that the one in PLRM mode. The SI and R are similar. The PLRM SWH is larger than NDBC buoys’ wave height when wave height is over 4 m, while SAR mode SWH does not show this characteristic. For Sentinel-3B, the bias, RMSE, SI and R of SAR and PLRM modes are similar. The PLRM SWH is also larger than NDBC buoys’ wave height when wave height is over 4 m. In general, Sentinel-3A/3B PLRM mode SWH is overestimated slightly when wave height is over 4 m. In conclusion, the accuracies of Sentinel-3A/3B SAR and PLRM mode SWH data are similar.

**Figure 11:**
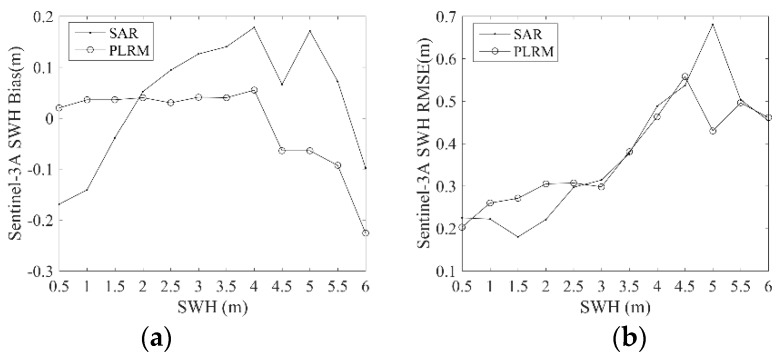
Dependence of (**a**) bias and (**b**) RMSE of Sentinel-3A SWH on buoy wave height.

**Figure 12:**
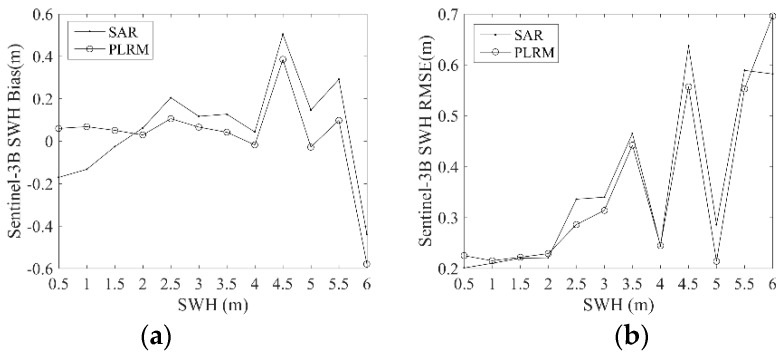
Dependence of (**a**) bias and (**b**) RMSE of Sentinel-3B SWH on buoy wave height.

In order to analyze the errors of Sentinel-3A/3B SWH at different wave heights, the biases and RMSEs between Sentinel-3A/3B and NDBC buoy data are calculated in bins of buoy wave heights of 0.5 m, when wave height is not more than 6 m. The results are shown in Figure 11 and Figure 12. The value at 0.5 m on the *x*-axis corresponds to the result of the collocated SWH data between 0 m and 0.5 m, and the rest of the values on this axis are displayed similarly. It is shown in Figure 11 that the bias of Sentinel-3A PLRM SWH is stable at first and then decreases rapidly when wave height is larger than 4 m. This means that Sentinel-3A SWH is underestimated when wave height is larger than 4 m. The bias of Sentinel-3A SAR mode SWH increases firstly and then decreases with increasing wave height. RMSEs of Sentinel-3A SAR and PLRM mode SWH increases with increasing wave height. Similar results are observed in Figure 12 for Sentinel-3B SAR and PLRM mode SWH. These results mean that the accuracy of Sentinel-3A/3B SWH data decreases with increasing wave height. In general, the PLRM mode SWH is slightly better than the SAR mode SWH.

**Figure 13:**
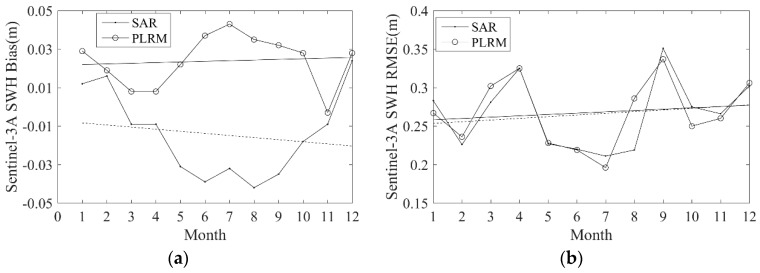
Monthly (**a**) bias and (**b**) RMSE of Sentinel-3A SWH in the different months of a year; the solid and dotted straight lines are the fitting trends.

For nearly 3 years (from 13 March 2016 to 25 February 2019), Sentinel-3A SWH data are collocated with NDBC buoy data, and the monthly bias and RMSE of Sentinel-3A SWH data over these 3 years are calculated and shown in Figure 13. The monthly bias of Sentinel-3A SAR mode SWH has an upward trend with a little fluctuation over the year. The monthly bias of Sentinel-3A PLRM mode SWH decreases between January and August and then increases. The monthly RMSEs of Sentinel-3A SAR and PLRM mode SWH have the same behavior over the year, and have a period of about half a year. These results imply that the Sentinel-3A SAR and PLRM mode SWH data have a similar accuracy over different months, and their errors show fluctuation over a period of about half a year.

**Figure 14:**
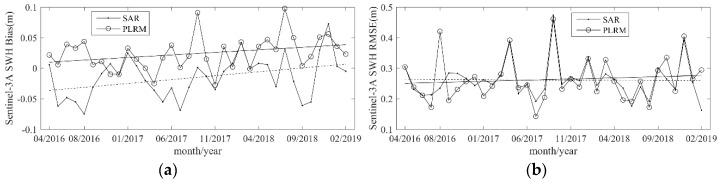
(**a**) Monthly bias and (**b**) RMSE of Sentinel-3A SWH across different months from February 2017 to January 2019; the solid and dotted straight lines are the fitting trends.

With a time series of almost 3 years of collocated Sentinel-3A and NDBC buoy data, the stability and drift of Sentinel-3A SWH data performance are analyzed. The monthly bias and RMSE of the collocated data from March 2016 to February 2019 are shown in Figure 14. It is shown that the monthly bias of Sentinel-3A SAR and PLRM mode SWH has a slight upward trend. The variations in the monthly RMSE of Sentinel-3A SAR and PLRM mode SWH are similar across different months, except for two large fluctuations in April and September 2017. These results show that the accuracies of Sentinel-3A SAR and PLRM mode SWH data show a slight decreasing trend over time.

### 3.2. Comparison with Jason-3 Data

**Figure 15:**
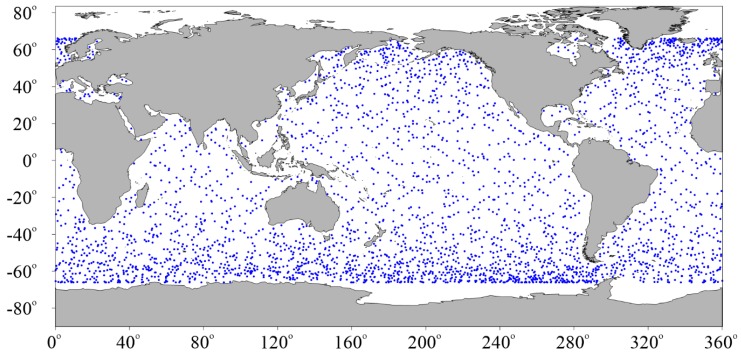
Spatial distribution of dual-crossovers of Sentinel-3A and Jason-3.

Although buoy data are the most commonly used field data to validate altimeter SWH data, the spatial distribution of buoys in the global oceans is sparse and spatially irregular, especially for the open sea areas. In order to validate Sentinel-3A SWH data on the global oceans with no buoy wave observations, Sentinel-3A SWH data are compared with Jason-3 data at their dual-crossovers within an observation time difference of less than 30 min in the same period. A total of 4250 data pairs of Sentinel-3A and Jason-3 at their dual-crossovers are collocated by the method introduced in Section 2.4. The collocated data are distributed all over the global oceans, as shown in Figure 15. For the period of Sentinel-3B, the data is too short (less than 4 months), so the Sentinel-3B SWH data are not compared with Jason-3.

**Figure 16:**
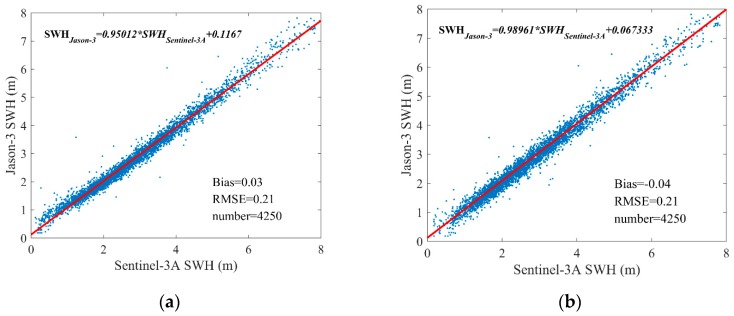
Scatterplots for the SWH comparison between Sentinel-3A (**a**) SAR and (**b**) PLRM modes and Jason-3 at their dual-crossovers.

**Figure 17:**
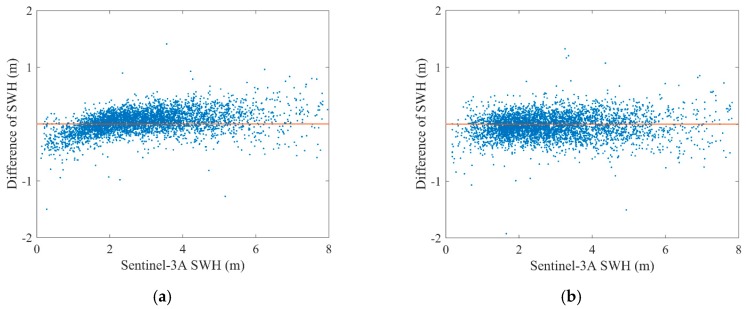
Dependence of SWH difference between Sentinel-3A (**a**) SAR and (**b**) PLRM modes SWH and Jason-3 at their dual-crossovers on the Sentinel-3A SWH.

The bias and RMSE of the SWH differences between Sentinel-3A and Jason-3 are calculated. The scatter diagrams of the comparison between Sentinel-3A SAR and PLRM mode SWH and Jason-3 data at their dual-crossovers and their regression expressions are shown in Figure 16. It is visible in Figure 16 that the Sentinel-3A PLRM mode SWH data are slightly more consistent with Jason-3 SWH data than those of Sentinel-3A SAR mode, and Sentinel-3A SAR mode SWH is slightly larger than that of Jason-3. The bias and RMSE of Sentinel-3A SAR and PLRM mode SWH are 0.03 m and 0.21 m, and −0.04 m and 0.21 m, respectively. The bias and RMSE of Sentinel-3A SAR and PLRM mode SWH data are similar. The results show that Sentinel-3A PLRM mode SWH data are slightly closer to those of Jason-3 than Sentinel-3A SAR mode, from the global ocean perspective. The differences in SWH between Sentinel-3A and Jason-3 versus Sentinel-3A SWH are shown in Figure 17. The SWH differences between Sentinel-3A PLRM mode SWH and Jason-3 fluctuate around 0 m. However, the SWH differences between Sentinel-3A SAR mode SWH and Jason-3 are slightly larger than 0 m and increase with increasing wave height. This also proves that Sentinel-3A PLRM mode SWH is more consistent with Jason-3 than SAR mode SWH over the global ocean.

### 3.3. Calibration of Sentinel-3A/3B SWH Data

The calibration of Sentinel-3A/3B SWH data is carried out by comparing Sentinel-3A/3B SWH data with the NDBC buoy data given in Section 3.1. Based on the collocated Sentinel-3A/3B SAR and PLRM mode SWH and NDBC buoy data, Sentinel-3A/3B SAR and PLRM mode SWH are adjusted to agree more closely with NDBC buoy data by using piecewise linear regression analysis. In this study, the Sentinel-3A/3B SWH data are taken as dependent and NDBC buoy data as independent variables. Then NDBC buoy-measured wave height (taken as the truth) can be written as the linear function of Sentinel-3A/3B SWH. The linear function is given as:

(5) $$SWH_{NDBC}=a\times SWH_{Sentinel-3}+b$$

where, *SWH_sentinel-3_* and *SWH_NDBC_* are the SWH of Sentinel-3A/3B and the wave height of NDBC buoys, and *a* and *b* are the undetermined coefficients of linear function.

**Figure 18:**
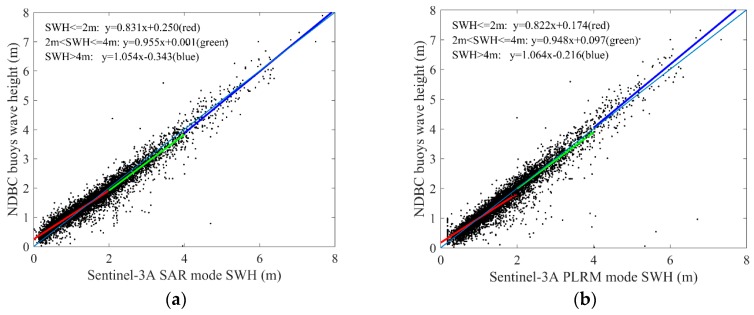
Calibration results of Sentinel-3A SAR (**a**) and PLRM (**b**) mode SWH data.

**Figure 19:**
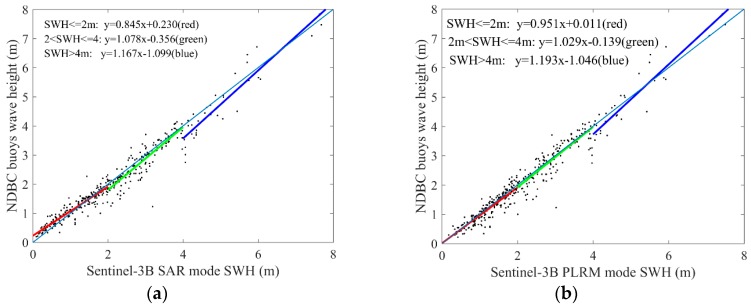
Calibration results of Sentinel-3B SAR (**a**) and PLRM (**b**) mode SWH data.

By analyzing the collocated data between Sentinel-3A/3B and the NDBC buoys, we select a three-pieces linear function to calibrate the Sentinel-3A/3B SWH data by dividing the NDBC buoys wave height into three parts, which are SWH ≤ 2 m, 2 m < SWH ≤ 4 m and SWH > 4 m (taken as low, middle and high level waves). The coefficients of the calibration functions and the statistical parameters of Sentinel-3A/3B SWH data before and after calibration are given in Table 2 and Table 3. The calibration results for Sentinel-3A and Sentinel-3B SWH data are shown in Figure 18 and Figure 19, respectively. For the calibration of Sentinel-3A SWH data, the accuracies of SAR mode SWH in the middle and high level waves are obviously improved, and the accuracies of PLRM mode SWH in the low and middle level waves are improved. In general, RMSE of Sentinel-3A SAR mode SWH is reduced from 0.269 m to 0.244 m by the calibration, and that of PLRM mode SWH is reduced from 0.297 m to 0.286 m. For the calibration of Sentinel-3B SWH data, the accuracies of SAR mode SWH and PLRM mode SWH in high level waves are improved largely. RMSE of Sentinel-3B SAR mode SWH is reduced from 0.312 m to 0.276 m, and that of PLRM mode SWH is reduced from 0.297 m to 0.285 m.

## 4. Conclusions

In this study, Sentinel-3A SWH data from 13 March 2016 to 25 February 2019 and Sentinel-3B SWH data from 10 November 2018 to 7 March 2019 are validated by the comparison of Sentinel-3A/3B SWH with NDBC buoy data, and the comparison of Sentinel-3A SWH data with Jason-3 data at their dual-crossovers. Through the influence analysis of spatial and temporal scales on the data collocation and validation of Sentinel-3A/3B and NDBC buoy data, the spatial scale of 25 km and the temporal scale of 30 min are selected to collocate 5840 data pairs with 172 NDBC buoys for Sentinel-3A, and 378 data pairs with 123 NDBC buoys for Sentinel-3B. The biases and RMSEs of Sentinel-3A SAR and PLRM mode SWH are −0.04 m and 0.27 m, and 0.03 m and 0.30 m, respectively. The Sentinel-3A SAR and PLRM mode SWH are consistent with NDBC buoy data, except for a little overestimation of buoy wave height by PLRM mode SWH when wave height is larger than 4 m. The biases and RMSEs of Sentinel-3B SAR and PLRM mode SWH are 0.05 m and 0.31 m, and 0.06 m and 0.30 m, respectively. The same results can be found for Sentinel-3B SWH as those found for Sentinel-3A SWH. The PLRM mode SWH is slightly better than that of SAR mode. The statistical analysis of Sentinel-3A/3B SWH in the bin of 0.5 m wave height shows that the accuracy of Sentinel-3A/3B SWH data decreases with increasing wave height. In order to investigate the long-term stability and drift of Sentinel-3A SWH, the monthly biases and RMSEs of Sentinel-3A SWH and NDBC data from March 2016 to January 2019 are analyzed, and the results show that the Sentinel-3A SWH are stable and show no major drift over time.

Due to the restricted geographical distribution of NDBC buoys, it is not possible to achieve validation results for SWH using NDBC buoys, from the perspective of the global ocean. So, a SWH comparison between Sentinel-3A and Jason-3 at their dual-crossovers, with the observation time difference at less than 30 min, is proposed to validate Sentinel-3A SWH in the global ocean. The comparison results show that the RMSE of SWH difference between Sentinel-3A and Jason-3 are no more than 0.21 m, and they are consistent in the global ocean, so the two modes of Sentinel-3A SWH have the same accuracy as Jason-3, except Sentinel-3A SAR mode SWH which overestimates Jason-3 slightly. With the collocated Sentinel-3A/3B and NDBC buoy data, Sentinel-3A/3B SWH data are calibrated by using piecewise linear regression analysis. The calibration functions of Sentinel-3A/3B SWH are given and the RMSE of Sentinel-3A/3B SWH is reduced by about 0.01–0.04 m. In general, Sentinel-3A/3B SWH data have high accuracy and remain stable. Sentinel-3A and -3B supply a new data resource of global ocean wave height.

## Figures and Tables

**Table 1:** Basic technical characteristics of Sentinel-3A/3B SAR Altimeter (SRAL) instrument [20,21].

Parameter	Ku Band	C Band
Frequency	13.575 GHz	5.41 GHz
Bandwidth	350 MHz	320 MHz
Tracking modes	closed-loop and open-loop	
Low-Resolution Mode (LRM) PRF	1920 Hz	
Synthetic Aperture Radar (SAR) mode PRF	18 kHz	
SAR along track resolution	~300 m	

**Table 2:** Coefficients of calibration functions and statistical parameters of Sentinel-3A SWH data.

Mode	SWH	a	b	Before Calibration		After Calibration	
				Bias (m)	RMSE (m)	Bias (m)	RMSE (m)
	SWH ≤ 2 m	0.831	0.250	−0.069	0.221	0.0	0.180
SAR	2 m < SWH ≤ 4 m	0.995	0.001	0.117	0.336	0.0	0.314
	SWH > 4 m	1.054	−0.343	0.076	0.557	0.0	0.550
	SWH ≤ 2 m	0.822	0.174	0.034	0.267	0.0	0.251
PLRM	2 m < SWH ≤ 4 m	0.948	0.097	0.037	0.334	0.0	0.331
	SWH > 4 m	1.064	−0.216	−0.090	0.505	0.0	0.493

**Table 3:** Coefficients of calibration functions and statistical parameters of Sentinel-3B SWH data.

Mode	SWH	a	b	Before Calibration		After Calibration	
				Bias (m)	RMSE (m)	Bias (m)	RMSE (m)
	SWH ≤ 2 m	0.845	0.230	0.050	0.215	0.0	0.190
SAR	2 m < SWH ≤ 4 m	1.078	0.356	0.131	0.352	0.0	0.324
	SWH > 4 m	1.167	1.099	0.259	0.537	0.0	0.439
	SWH ≤ 2 m	0.951	0.011	0.050	0.223	0.0	0.216
PLRM	2 m < SWH ≤ 4 m	1.029	−0.139	0.057	0.325	0.0	0.319
	SWH > 4 m	1.193	−1.046	0.104	0.497	0.0	0.448
